# Microbiology Meets Archaeology: Soil Microbial Communities Reveal Different Human Activities at Archaic Monte Iato (Sixth Century BC)

**DOI:** 10.1007/s00248-016-0904-8

**Published:** 2016-12-13

**Authors:** Rosa Margesin, José A. Siles, Tomas Cajthaml, Birgit Öhlinger, Erich Kistler

**Affiliations:** 1 Institute of Microbiology, University of Innsbruck, Technikerstrasse 25, 6020 Innsbruck, Austria; 2 Institute of Microbiology, Academy of Sciences of the Czech Republic, v.v.i., Vídeňská 1083, 142 20 Prague 4, Czech Republic; 3 Institute for Environmental Studies, Faculty of Science, Charles University in Prague, Benatska 2, 128 01 Prague 2, Czech Republic; 4 Institute of Archaeologies, University of Innsbruck, Langer Weg 11, 6020 Innsbruck, Austria

**Keywords:** Archaeomicrobiology, Soil bacterial and fungal diversity, Culturable and nonculturable bacteria, PLFA, Community level physiological profile (CLPP)

## Abstract

Microbial ecology has been recognized as useful in archaeological studies. At Archaic Monte Iato in Western Sicily, a native (indigenous) building was discovered. The objective of this study was the first examination of soil microbial communities related to this building. Soil samples were collected from archaeological layers at a ritual deposit (food waste disposal) in the main room and above the fireplace in the annex. Microbial soil characterization included abundance (cellular phospholipid fatty acids (PLFA), viable bacterial counts), activity (physiological profiles, enzyme activities of viable bacteria), diversity, and community structure (bacterial and fungal Illumina amplicon sequencing, identification of viable bacteria). PLFA-derived microbial abundance was lower in soils from the fireplace than in soils from the deposit; the opposite was observed with culturable bacteria. Microbial communities in soils from the fireplace had a higher ability to metabolize carboxylic and acetic acids, while those in soils from the deposit metabolized preferentially carbohydrates. The lower deposit layer was characterized by higher total microbial and bacterial abundance and bacterial richness and by a different carbohydrate metabolization profile compared to the upper deposit layer. Microbial community structures in the fireplace were similar and could be distinguished from those in the two deposit layers, which had different microbial communities. Our data confirmed our hypothesis that human consumption habits left traces on microbiota in the archaeological evidence; therefore, microbiological residues as part of the so-called ecofacts are, like artifacts, key indicators of consumer behavior in the past.

**Electronic supplementary material:**

The online version of this article (doi:10.1007/s00248-016-0904-8) contains supplementary material, which is available to authorized users.

## Introduction

Human activity influences soil properties and is accompanied by the input of specific organic materials into soil. After the removal of the anthropogenic impact, organic matter is lost due to mineralization and transformation by soil microorganisms. However, the ancient anthropogenic impact on soils can be preserved in soil microbiota and their activities [1–3]. High input of organic matter usually stimulates microbial activity, which leads to increased microbial biomass and enzyme activity. There is evidence that paleosols in the surroundings of archaeological monuments, such as habitation layers of ancient settlements, bear the record not only of the environmental conditions of the past but also of the life of ancient people [1, 2, 4].

Soil studies at archaeological sites have a long history, but most of them focus on general aspects of soil chemistry, in particular pH variation, microelements, or micromorphology. In recent years, however, soil studies related to archaeological projects in Russia and elsewhere have introduced a microbiological perspective to the investigation of paleosols and sediments that had been anthropogenically influenced [3, 4]. The use of microbiology as a tool within nonbiological fields provides valuable data to complement and to upgrade knowledge about past events. For example, on a geological scale, microbiological data can contribute to the understanding and reconstruction of past climatological, environmental, and sedimentary conditions. Moreover, on the time scales of human history and archaeology, microbiological data can contribute to unraveling human cultural habits, microbe-related human diseases, and alterations in cultural artifacts due to biochemical reactions [5]. For example, microbiological studies in archaeological contexts focused on pathogens and on the abundance of microbial spores or coprophilous microorganisms ([3] and references therein).

Since 2011, archaeological research on Monte Iato in Italy has focused on cultural contacts in Archaic Western Sicily (eighth to sixth centuries BC). Specifically, the aim is to investigate more closely the effects of intercultural links and trans-Mediterranean interrelationships on the formation and transformation of local identities and elites [6, 7]. This is currently being tackled by means of a multimethodological approach that includes, alongside fine stratigraphic exploration, also archaeometric examination of early Iron Age finds on Iato [8–10].

Archaeological assemblages are the result of consumption-related events. As a result of human behavior, artifacts (metals, ceramics, glass, etc.) and bioarchaeological residues (animal bones, seeds, microorganisms, etc.) come together in close proximity to form a closed association of things in the archaeological record [11]. In the American tradition, in which archaeology is one of the four subdisciplines of anthropology, bioarchaeological remains are often understood as nonartifactual data and environmental factors—therefore called ecofacts—which significantly influenced human behavior and communal life at a given time and certain place [12, 13]. Later, they were rather understood as biological residuals, which are indeed caused by nature, but they have found their way into archaeological layers only through human activities [14]. In this respect, “ecofacts” are, as well as artifacts, key indicators of human behaviors, especially in the areas of consumption-related events.

Archaeological investigations of Archaic Iato in recent years resulted in a selective flotation of archaeological assemblages that promised detailed answers to questions about consumer habits and the local appropriation of global goods due to their composition and their topographical context (Forstenpointner and Weissengruber, personal communication; Thanheiser, personal communication). Generally, the archaeologies of the Mediterranean became more and more aware of the need for a “microarchaeology: beyond the visible archaeological record” [15] in recent years. Consequently, the desire arose to investigate also those bioarchaeological remains in the archaeological record on Monte Iato that are not visible to the naked eye. Microbiological analysis promised major insights, especially in terms of an “integrative archaeology” [16]. Therefore, the objective of this pilot study was the first examination of soil microbial communities in the anthropogenic layers of a (indigenous) residential building of the sixth century BC at Monte Iato. Soil samples from four sampling locations were collected from different archaeological layers: (1) the upper and the lower infill of a ritual deposit (food waste disposal) in the main room (sampling locations A6, A7) and (2) the strata above the fireplace in the annex (sampling locations A2, A3). Microbial characterization included abundance, activity, diversity, and community structure of soil bacteria and fungi. This is one of the first comprehensive surveys of bacterial and fungal communities, using both culture-based and nonculture-based techniques, to characterize archaeological strata as consumer-produced assemblages from a microbiological viewpoint.

## Materials and Methods

### Site Description and Soil Sampling

Monte Iato is situated about 30 km south-west of Palermo in Sicily, Italy (37° 58′ 01.9″ N, 13° 11′ 44.0″ E). Due to the advantages of this central position in terms of travel and transport routes, Monte Iato was settled during the early first millennium BC; by the late sixth and early fifth centuries BC, the settlement had become one of the most important indigenous centers of the West Sicilian interior [8, 17]. After a period of decline, it flourished once again in the early third century, the indigenous settlement acquiring the appearance of a Hellenistic polis together with the name Iaitas ([8] and references therein). After various phases of political and ethnic upheaval, the ancient city developed into medieval Giato, which, as the last refuge of the West Sicilian caliphate, was destroyed in 1246 AD by Frederick II [7, 18].

**Fig. 1:**
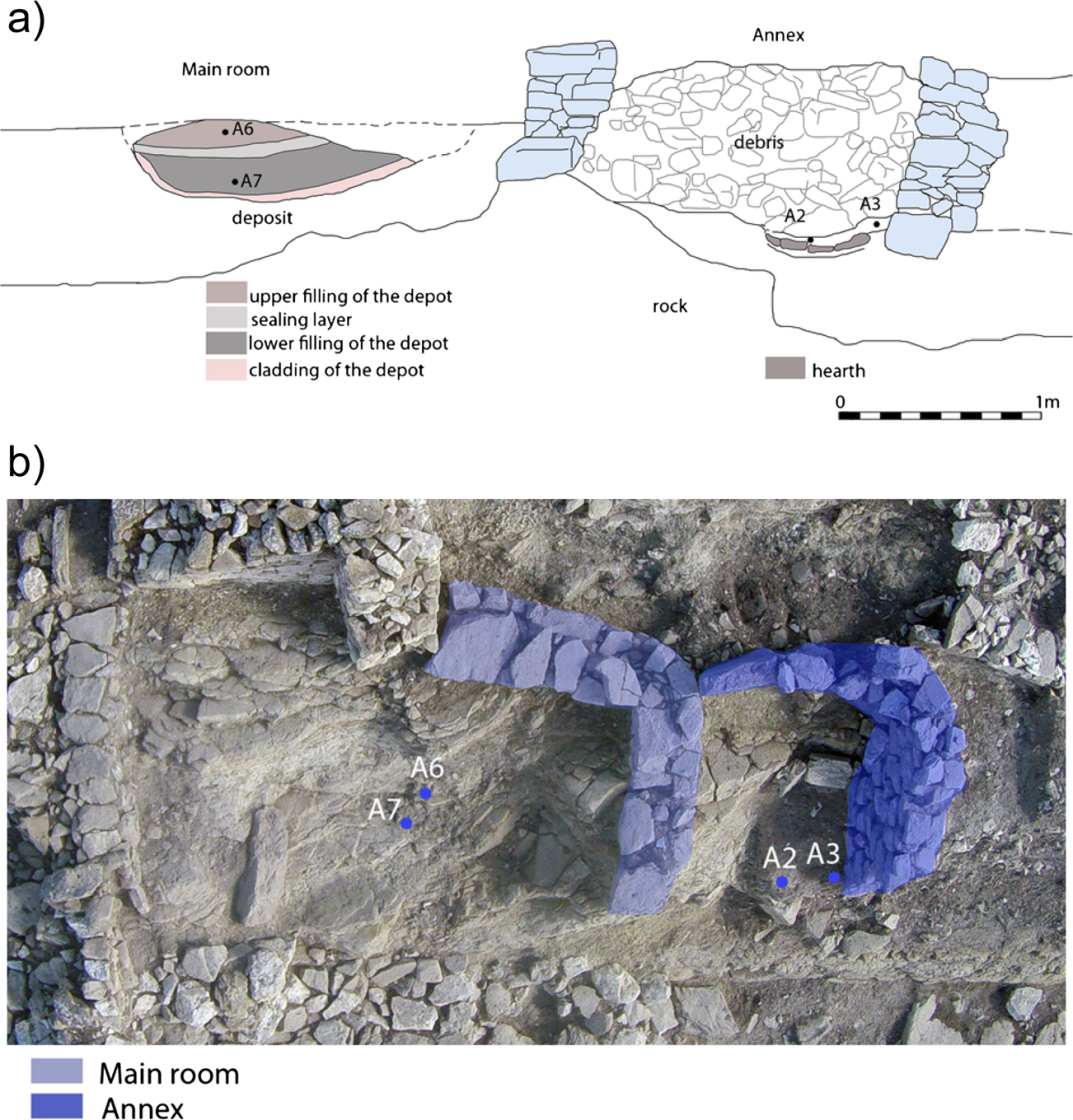
Sketch of the N-profile of the native building with indication of the sampling locations (**a**). Bird’s eye view of the excavation area of the native building (**b**) (Institute of Archaeologies, University of Innsbruck)

During the excavation of the Late Archaic House (Fig. S1B), about 20 m west of the so-called Aphrodite Temple (Fig. S1A), the first remains of the native (indigenous) residential building came to light in 2006 (37° 58′ 02.2″ N, 13° 11′ 43.3″ E) (Fig. 1, Fig. S1C). These were covered by several backfills and settlement layers of post-Archaic times (0.90 m to turf). In 2014, it became apparent that the native building consisted of a quadrangular main room and a rounded annex. The construction of this building cannot be dated more accurately than in the sixth century BC. The final abandonment and ritual destruction took place shortly before 500 BC as the Late Archaic House (Fig. S1B) was built to replace the old dwelling (Fig. S1C). For this purpose, the walls of the annex were brought to collapse and the remaining walls were filled with stones up to the top edge (Fig. 1) [7, 19]. In contrast to the annex, no debris was found in the main room (Fig. 1). Instead, a pit was dug into the floor and the foundations, lined with a layer of stones. On top of the packing and in the immediate vicinity antlers of deer, a fragment of a ram-skull with horn core and the horn core of an ox have been found, which are clear traces of cultic activities [20]. The archaeological record of this indigenous building is therefore connected to a concentrated materialization of various social events (everyday use, ritual abandonment, and cultic reuse) [20].

Under the debris of the annex walls and 32 cm under the floor of the main room (top edge 829.73 m above sea level), the burnt clay of a hearth was found. In a second phase, this fireplace has been lined with mainly nonmatching ceramic sherds of two thick-walled vessels. Soil samples were collected in September 2014 directly above the hearth under the stones of the debris within an area of 0.5 × 0.5 m (sampling locations A2, A3) (Fig. 1).

The deposit, respectively the pit in the main room, was filled with two rubbish layers, which contained ceramics and bones. The lower infill was sealed with a solid and compact layer of clay and calcareous pebble, while the upper layer was covered by a stratum of stones. Both the upper layer (sampling location A6; 10 cm under the floor of the main room) and the lower infill (sampling location A7; 28 cm under A6) of the deposit were sampled in September 2014 within an area of 1.65 × 1 m (Fig. 1).

At each sampling location, soil was sampled from five sampling spots to cover within-site variability and placed in sterile vials by using sterile spatulas. Immediately after sampling, soil samples were stored in cooled boxes, shipped to the laboratory in Innsbruck, immediately processed, or stored at 2 °C under aerobic conditions for microbial activity measurements, or at -80 °C prior to phospholipid fatty acid (PLFA) and DNA analyses. Due to the low amount of soil per sample, allowing only few analyses, we produced composite soil samples (that were analyzed in triplicate) for each of the sampling locations under sterile conditions immediately before analysis. This way we were able to apply a wide spectrum of methods in order to gain a first overview of microbial characteristics at the four sampling locations (A2, A3, A6, A7).

### Physicochemical Soil Characterization

Analysis of soil samples included measurements of dry mass (105 °C), pH (CaCl_2_), contents of soil organic matter (loss on ignition at 600 °C), total organic carbon (TOC), and total nitrogen, following standard methods [21]. C/N ratio was calculated as TOC/N. Heavy metal contents were determined after digestion with 65% (*w*/*v*) HNO_3_; Ag, Co, Cr, Ca, Fe, Ni, and Pb were measured by atomic absorption spectrometry. All results were calculated on a soil dry mass basis.

### Phospholipid Fatty Acid Analysis

PLFA from soil samples were extracted using a mixture of chloroform-methanol-phosphate buffer (1:2:0.8; *v*/*v*/*v*) according to Bligh and Dyer [22]. Phospholipids were then separated using solid-phase extraction cartridges (LiChrolut Si-60, Merck, Whitehouse Station, USA) and subjected to mild alkaline methanolysis as described [23]. The free methyl esters of phospholipid fatty acids were analyzed by gas chromatography mass spectrometry (450-GC, 240-MS ion trap detector, Varian, Walnut Creek, USA) as described [24]. The PLFA i14:0, i15:0, a15:0, 16:1ω9, 16:1ω7, 16:1ω5, 10Me-16:0, i17:0, a17:0, cy17:0, 17:0, 10Me-17:0, 10Me-18:0, and cy19:0 were addressed as bacterial markers. The PLFA 10Me-16:0, 10Me-17:0, and 10Me-18:0 were used as actinobacterial markers. The fatty acid 18:2ω6,9 was selected as fungal marker. The fatty acids found both in bacteria and fungi, such as 15:0, 16:0, and 18:1ω7, were excluded from the analysis [25]. The total content of PLFA was used as a measurement of total microbial biomass. Ratios between PLFA markers derived from fungi and bacteria were calculated [26].

### Community Level Physiological Profiling

The Biolog EcoPlate system (BIOLOG Inc., CA, USA) was used to determine community level physiological profiles (CLPP) for each sampling location. Each Biolog EcoPlate contains 31 different kinds of carbon sources (ten types of carbohydrates, nine carboxylic/acetic acids, six amino acids, four polymers, and two amines/amides) [27]. The analyses were performed in triplicate. To determine the CLPP for each sample, 1 g of soil fresh mass was shaken in 10 ml of sterile saline solution (0.85% *w*/*v* NaCl) at 150 rpm for 1 h [28, 29]. Soil suspensions were then serially diluted to 10^−4^ based on pretesting. Soil solutions (130 μl) were used for each well and Ecoplates were incubated at 20 °C for 14 days. Color development in each well was recorded as optical density (OD) at 590 nm every 24 h using an automated plate reader. Microbial activity was calculated as average well color development (AWCD) as described [29]. OD values after 10 days were normalized by dividing them by their AWCD and were selected to determine the relative consumption rate of each substrate and C-source family.

### Soil Microbial Community Structure and Diversity: Culture-Independent Approach

#### DNA Extraction

Total DNA was extracted in triplicate from each sampling location from 250 mg of soil fresh mass, using the Power Soil™ DNA Isolation Kit (MoBio Laboratories Inc.). Subsequently, all DNAs were quantified using QuantiFluor™ dsDNA System (Promega) and the DNA concentration for each extraction was standardized to 5 ng μl^−1^.

#### 16S rRNA Gene Fragment and ITS1 Region Amplification and Sequencing

For bacteria, a fragment of 16S ribosomal RNA (rRNA) gene was amplified using the primers 27F and 519R [30], capturing V1-V3 regions. Three different DNA extractions from each sampling location were amplified using the HotStarTaq Plus Master Mix Kit (Qiagen) containing barcoded forward primers, under the following thermal conditions: (i) initial denaturation at 94 °C for 3 min; (ii) 28 cycles of denaturation at 94 °C for 30 s, primer annealing at 55 °C for 40 s, and extension at 72 °C for 60 s; with (iii) a final elongation at 72 °C for 5 min. PCR products were then purified using calibrated AMPure XP beads (Beckman Coulter, Inc.) and combined in equimolar ratios. Subsequently, the pooled and purified product was used to prepare a DNA library following the Illumina TruSeq DNA library preparation protocol. Paired-end sequencing (2 × 300) was performed on the Illumina MiSeq sequencing platform (Illumina) at MR DNA (www.mrdnalab.com, Shallowater, TX, USA). In the case of fungi, internal transcribed spacer (ITS) 1 region was firstly amplified using the ITS1 and ITS2 primers [31], with the following cycling parameters: (i) 94 °C for 3 min; (ii) 28 cycles of 94 °C for 30 s, 57 °C for 40 s, and 72 °C for 60 s; and (iii) a final elongation step at 72 °C for 5 min. Secondly, the processing of PCR products for paired-end sequencing (2 × 300) on Illumina MiSeq sequencing platform was done as described for bacteria.

#### Processing of Illumina Data

First, raw Illumina MiSeq reads were assembled and reoriented using MR DNA pipeline for bacterial 16S rRNA and fungal ITS1 libraries. Sequences were then demultiplexed and formatted for processing using a Phython script (http://drive5.com/usearch/manual/uparse_pipeline.html). Next, bacterial sequences were quality-filtered and clustered into operational taxonomic units (OTUs) using UPARSE pipeline and UPARSE algorithm [32]. Briefly, sequences were quality-filtered allowing a maximum e-value of 0.5. Subsequently, reads were trimmed to 440-bp (base pairs) length as well as dereplicated and sorted by abundance, removing singletons (sequences which appeared once) with prior OTU determination at 97% sequence identity. Then, putative chimeric OTUs were removed using UCHIME [33] and gold database as reference. Abundance data were then reincorporated into the dataset by mapping the initial sequences against the representative OTUs and one OTU table was generated. In the case of fungal sequences, they were quality-filtered and dereplicated and singletons were removed as bacterial process. Next, ITS1 region was extracted using ITSx software [34]. Sequences were trimmed to 140 bp and grouped in OTUs at 97% identity as bacterial sequences. Chimeric OTUs were removed and an OTU table was generated. The taxonomic affiliation of each OTU was obtained using Ribosomal Database Project taxonomic classifier [35] against 16S rRNA training set 10 for bacterial sequences and UNITE Fungal ITS trainset 07-04-2014 for fungal sequences using an 80% threshold for bacteria and a 50% threshold for fungi [36].

Except for rarefaction curves, the number of sequences per sample was normalized to 20,000 sequences for bacteria and to 25,000 sequences for fungi. The bacterial and fungal communities were characterized in terms of diversity by calculating richness (number of OTUs), Shannon index, Smith-Wilson evenness, and the richness estimator ACE (abundance-based coverage estimation) using Mothur v.1.34.4 [37].

#### Data Accessibility

The sequence data were deposited in the MG-RAST public database (http://metagenomics.anl.gov/) under accession number 4697523.3 for bacterial sequences and 4697524.3 for fungal sequences.

### Enumeration of Culturable Heterotrophic Soil Bacteria

Culturable heterotrophic soil bacteria were enumerated by the plate-count method for viable cells in triplicate as described [38]. Briefly, soil suspensions were prepared by shaking 1 g of soil fresh mass with 9 ml of sterile pyrophosphate solution (0.1% *w*/*v*) for 20 min. Appropriate dilutions of soil suspensions were surface spread onto R2A plates (0.05% yeast extract, 0.05% peptone, 0.05% casamino acids, 0.05% glucose, 0.05% starch, 0.03% sodium pyruvate, 0.03% K_2_HPO_4_, 0.005% MgSO_4_, 1.5% agar) containing cycloheximide (400 μg ml^−1^) to exclude fungal growth. Colony-forming units (CFU) were counted after 10 days of incubation at 20 °C and calculated on a soil dry mass basis.

### Isolation and Identification of Isolated Soil Bacteria

Fifty colonies from each sampling location were randomly selected and purified. Bacterial genomic DNA was extracted according to Marmur [39]. The primers 27F and 1541R [40] were used for partial 16S rRNA gene amplification. Each 50 μl PCR reaction contained 5 μl PCR buffer (10×) (BIORON GmbH, Germany), 1 μl MgCl_2_ (100 mM) (BIORON GmbH), 1 μl dNTPs (10 mM each) (Sigma, USA), 2 μl each of forward and reverse primers (10 μM) (VBC Biotech, Austria), 0.5 μl Taq polymerase (5 U μl^−1^) (BIORON GmbH), 4 μl DNA solution, and 34.5 μl H_2_O. The thermal cycling program was as follows: (i) 5 min at 95 °C; (ii) 35 cycles of 30 s at 95 °C, 30 s at 52 °C, and 1 min at 72 °C; and (iii) a final elongation step of 10 min at 72 °C. PCR products were purified using GENEJET PCR purification kit (Thermo Fisher Scientific Inc., USA), and the cleaned 16S rRNA PCR gene products were then sequenced (Macrogen, Amsterdam, Netherlands).

The sequences were edited with ClustalW implemented in the MEGA ver. 6 software, and the nearest phylogenetic neighbors were determined for each strain using the EzTazon-e Database [41]. This information was used to describe the taxonomic bacterial diversity. The sequences were then clustered into OTUs at 97% identity using Mothur, and one representative isolate per OTU was selected (Table S6) (GenBank database accession numbers KX028737 to KX028763). Subsequently, the strains representing each culturable OTU were phylogenetically compared (97% identity) with the sequences representing the top 32 most abundant OTUs obtained from nonculture-based Illumina analysis (Table S3).

### Enzyme Activities of Culturable Soil Bacteria

Activities of amylase, protease, cellulase, and lipase-esterase were tested for 50 isolates from each sampling location as described [42] on R2A agar supplemented with starch, skim milk (each compound 0.5% *w*/*v*), carboxymethylcellulose and trypan blue (0.5% and 0.01% *w*/*v*, respectively), or Tween 80 and CaCl_2_ (0.5% *v*/*v* and 0.01% *w*/*v*, respectively). Plates were incubated up to 5–12 days at 20 °C.

### Statistical Analyses

The pattern of C sources consumption at the four sampling locations was compared by principal components analysis (PCA) using CANOCO 5.0 for Windows, and the significance of the observed differences was determined by permutational multivariate analysis of variance (PERMANOVA) and analysis of similarity (ANOSIM) with Bray-Curtis similarities after 9999 permutations using PAST ver. 3.07 software [43]. Statistical differences between soil physicochemical and microbial characteristics (biomass, CLPP, diversity parameters, relative abundance of bacterial and fungal phyla and classes as well as the most abundant OTUs) were analyzed by ANOVA. Tukey’s honest significance difference (HSD) test was used for multiple comparisons of means at a 95% confidence interval after testing of the normality of data using the Shapiro-Wilk test.

Differences in the structure of nonculturable bacterial and fungal communities at the four sampling locations were assessed by PERMANOVA and ANOSIM (Bray-Curtis similarities after 9999 permutations). Compositions of bacterial and fungal communities were compared by UPGMA (also known as average linkage method) dendrogram considering OTU distribution using pvclust package in R [44]. The number of shared and specific OTUs at each sampling location was visualized using the Mothur ‘venn’ command.

## Results

### Soil Physicochemical Properties

Soils from all four sampling locations contained sand, silt, and a low amount of clay and had a pH in the range of 5.9–6.0. Due to the Fe-containing silicate bedrock (rubification), soils contained a high amount of Fe (11.2–17.0 g/kg soil dry mass). Generally, soil C and N contents were low. Soils from sampling locations A6 and A7 were characterized by significantly higher contents of nutrients than soils from A2 to A3: SOM and TOC contents were 1.7-fold higher and total nitrogen and the resulting C/N ratio were 1.3-fold higher at A2 and A3 (Table 1).

**Table 1:** Soil physicochemical properties and soil microbial biomass at the four sampling locations. For each variable, data followed by different letters are significantly different (*p* ≤ 0.05)

Parameter	A2	A3	A6	A7
pH (CaCl_2_)	5.9 a	6.0 a	6.0 a	6.0 a
SOM (%)	1.53 a	1.62 a	2.67 b	2.57 b
TOC (%)	0.89 a	0.94 a	1.55 b	1.49 b
N (%)	0.06 a	0.06 a	0.08 b	0.08 b
C/N	14.8 a	15.7 a	19.4 b	18.7 b
Heavy metals (mg/kg soil dry mass)				
Ag	0.3	0.1	0.1	0.2
Co	0.5	0.5	0.7	0.7
Cr	8.1	11.3	9.8	11.5
Cu	18.3	17.8	16.0	20.1
Fe	11,200	13,500	14,200	17,000
Ni	11.6	12.8	13.0	14.0
Pb	10.3	9.4	13.3	11.5
PLFA (ng/g soil dry mass)				
Total biomass	0.38 a	0.81 a	1.63 a	10.08 b
Bacteria	0.20 a	0.51 a	1.46 a	9.15 b
Actinobacteria	0.00 a	0.15 a	0.00 a	4.11 b
Fungi	0.10 a	0.13 a	0.17 a	0.13 a
Fungi/bacteria	0.84 a	0.46 a	0.81 a	0.01 a
Culturable bacteria				
CFU/g soil dry mass	9.1 × 10^6^ b	4.1 × 10^6^ ab	1.5 × 10^6^ a	2.5 × 10^6^ a

### Microbial Biomass: PLFA

PLFA analysis demonstrated the presence of living bacterial and fungal communities at all four sampling locations. Total microbial PLFA and PLFA of bacteria and Actinobacteria were significantly higher at A7 than at the other three locations. Neither fungal PLFA nor the ratio between fungal and bacterial PLFA was significantly different at the four sampling locations (Table 1).

### Enumeration of Culturable Heterotrophic Bacteria

Culturable bacteria at the four sampling locations were in the range of 1.5 × 10^6^ to 9.1 × 10^6^ cfu g^−1^ soil dry mass. In contrast to PLFA-related bacterial biomass, numbers of culturable bacteria at A6 and A7 were not significantly different ((2–3) × 10^6^ cfu g^−1^ soil) and were 2–3-fold lower than those at A2 and A3 (Table 1).

### Community Level Physiological Profiles

**Fig. 2:**
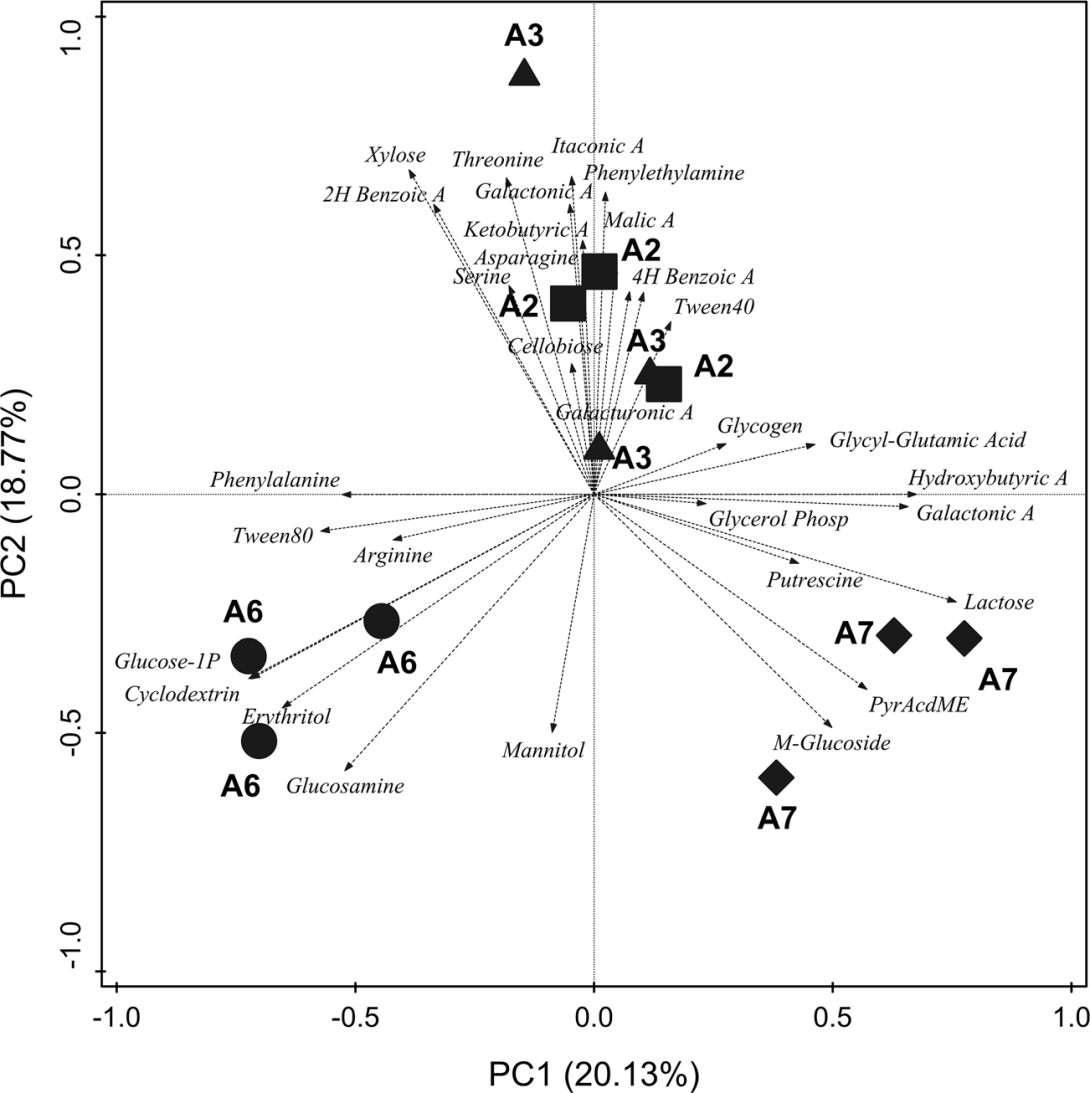
Biplot principal components analysis (PCA) of community level physiological profiles (CLPP) at the four sampling locations (A2, A3, A6, A7). Percent variability explained by each principal component is shown in *parentheses* in the axes legends

Biplot PCA of CLPP of the four sampling locations, with the two principal components explaining ca. 39% of the variability, clearly separated the sampling locations in three different clusters (Fig. 2), and PERMANOVA (*p* = 0.0006) as well as ANOSIM (*p* = 0.0008) analyses confirmed the significance of this ordination. One of the clusters was situated in the upper quadrants and comprised A2 and A3. Microorganisms in soils from these two sampling locations were characterized by a significantly higher ability to oxidize carboxylic and acetic acids (2-hydroxy benzoic acid, itaconic acid, d-galactonic acid-γ-lactone, 4-hydroxy benzoic acid, malic acid, α-ketobutyric acid) than microorganisms in soils from A6 to A7. In contrast, carbohydrates were consumed to a significantly higher extent in soils from A6 to A7 than in soils from A2 to A3 (Table S1). PCA demonstrated that the profile of carbohydrates metabolized by microorganisms at A6 (glucose-1-phosphate, erythritol, glucosamine) differed from that of A7 (β-methyl-d-glucoside, pyruvic acid methyl ester, α-d-lactose) (Fig. 2). Regarding the utilization of polymers, amino acids, and amines/amides, no quantitative differences in microbial oxidation rates between the four sampling locations were detected (Table S1). However, PCA showed that the patterns of amino acids and polymers oxidized at A2 and A3 differed from those at A6 and A7 (Fig. 2).

### Enzyme Activities of Culturable Bacteria

Culturable heterotrophic bacteria from all four sampling locations showed activities of protease (35–72% of ca. 50 isolates per site), amylase (18–47%), and lipase-esterase (15–44%), while cellulase activity was only detected with bacteria isolated from A6 to A7 (10–13%).

### Microbial Community Structure and Diversity: Culture-Independent Approach

Three different bacterial and fungal amplicon Illumina sequencing analyses were performed for microbial community evaluation at each sampling location. After analysis of data, it resulted that one of the three replicates per location failed due to an extremely low number of sequences or the distribution of sequences into a very small number of OTUs (in some cases, the total number of sequences was distributed among only two OTUs). Therefore, we rejected these replicates for further analysis and the described differences between bacterial and fungal communities at the four sampling locations are based on two replicates per sampling locations. Preinvestigations demonstrated good agreement between the results obtained from the analysis of two and three replicates, respectively.

#### Bacterial Communities

**Fig. 3:**
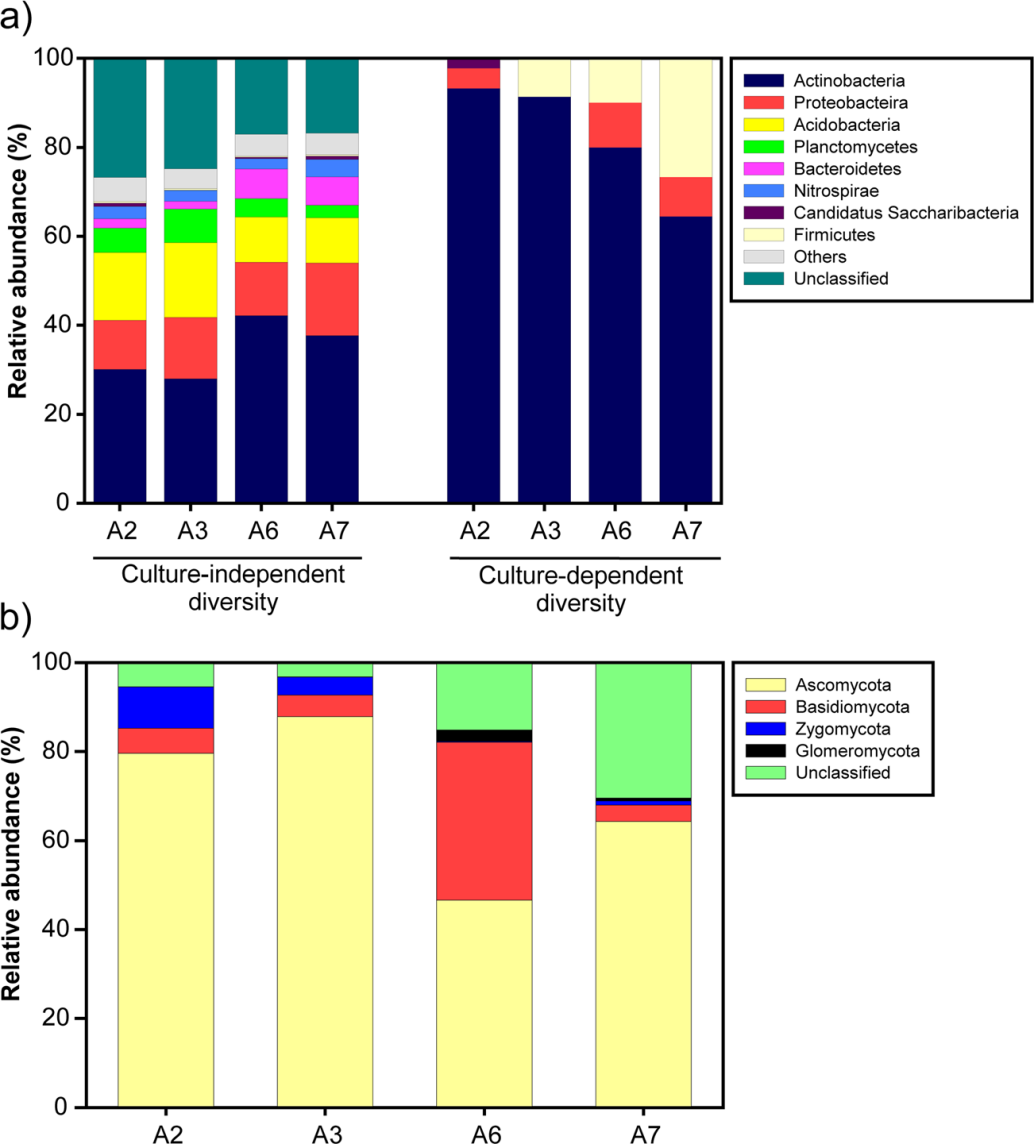
Relative abundance of nonculture-based and culturable bacterial (**a**) and nonculture-based fungal (**b**) phyla found at the sampling locations A2, A3, A6, and A7

Across the eight DNA extracts (two replicates per sampling location), we obtained a total of 201,243 high-quality bacterial 16S rRNA gene sequences with an average read length of 440 bp. The average number of sequences per sample was 25,125, ranging from 21,678 to 28,902. The total number of sequences clustered into 1466 OTUs at a 97% identity threshold, and all the OTUs could be classified as bacteria using an 80% threshold. The rarefaction curves demonstrated that this number of reads covered most of the community at each site (Fig. S2); 78.7% of total sequences were classified at the phylum level and 40–55% of the bacteria identified at the phylum level could be assigned to Gram-positive bacteria. At the phylum level, the overall bacterial community at the four sampling locations was dominated by sequences assigned to the Actinobacteria (with the number of classified sequences in this phylum ranging from 28.0 to 42.1%), Proteobacteria (11.0–16.4%), Acidobacteria (10.1–16.8%), Planctomycetes (2.9–7.6%), and Bacteroidetes (1.7–6.6%) (Fig. 3a). At the class level, 72.5% of the reads could be assigned, and the most abundant classes were *Actinobacteria* (with *Rubrobacteridae*, *Actinobacteridae*, and *Acidimicrobidae* as the most abundant orders in this subclass); *Alphaproteobacteria*, *Deltaproteobacteria*, and *Gammaproteobacteria* among Proteobacteria; subgroups 6, 4, and 7 in Acidobacteria; Bacteroidetes incertae sedis and *Sphingobacteriia* in Bacteroidetes and *Planctomycetia* in Planctomycetes (Fig. S3a).

Richness and richness estimator ACE significantly varied (*p* < 0.05) between the four sampling locations, with A2 showing the lowest values and A7 presenting the highest ones (Table 2). However, Shannon diversity index and evenness did not significantly vary between the four sampling locations (*p* > 0.05).

**Table 2:** Diversity characteristics of nonculturable bacterial and fungal communities at the four sampling locations A2, A3, A6, and A7. For each variable and microbial community, data followed by different letters are significantly different (*p* ≤ 0.05)

	A2	A3	A6	A7
Bacterial community				
Richness	784 a	894 ab	850 ab	950 b
Shannon index	5.10 a	5.39 a	5.20 a	5.32 a
ACE	938 a	1085 bc	989 ab	1127 c
Evenness	0.43 a	0.43 a	0.41 a	0.43 a
Fungal community				
Richness	107 a	115 a	120 a	124 a
Shannon index	165 a	133 a	180 a	136 a
ACE	2.30 a	2.61 a	2.47 a	2.53 a
Evenness	0.54 a	0.54 a	0.54 a	0.53 a

**Fig. 4:**
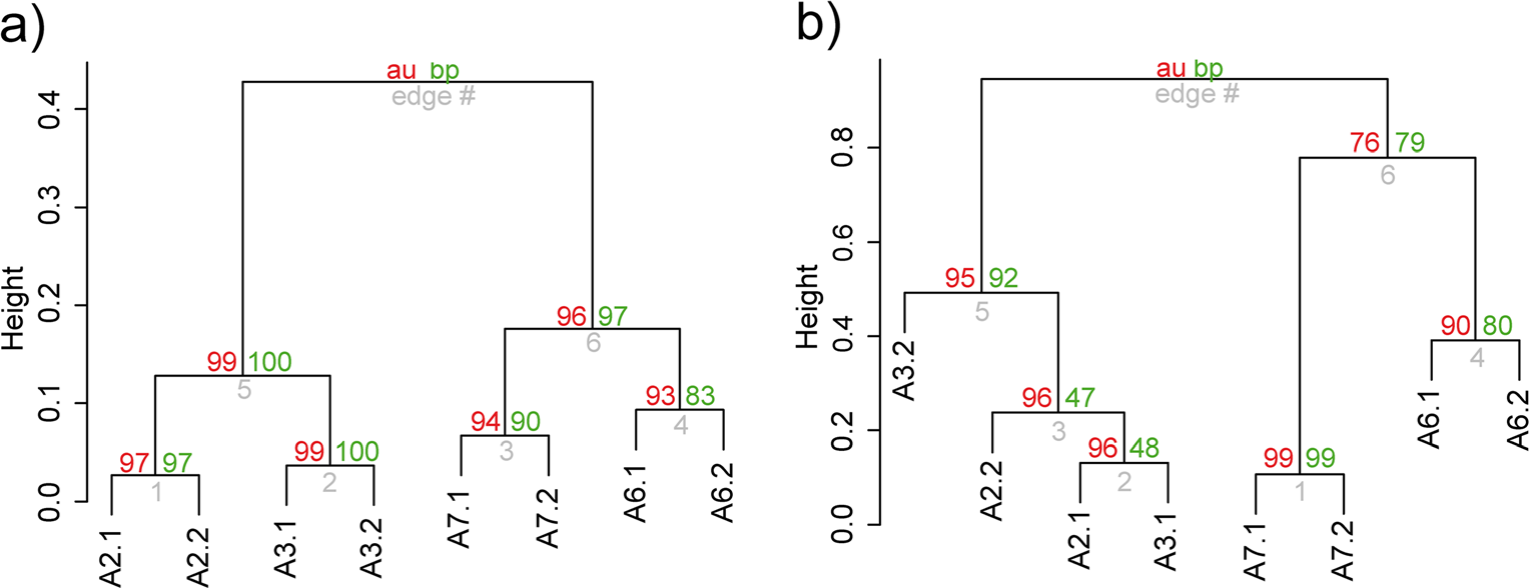
Cluster analysis based on the distribution of nonculture-based bacterial (**a**) and fungal (**b**) OTUs at the four sampling locations (A2, A3, A6, A7), generated by UPGMA. Values at branches are AU (approximately unbiased) *p* values (*left*), BP (bootstrap probability) values (*right*), and cluster labels (*bottom*)

**Fig. 5:**
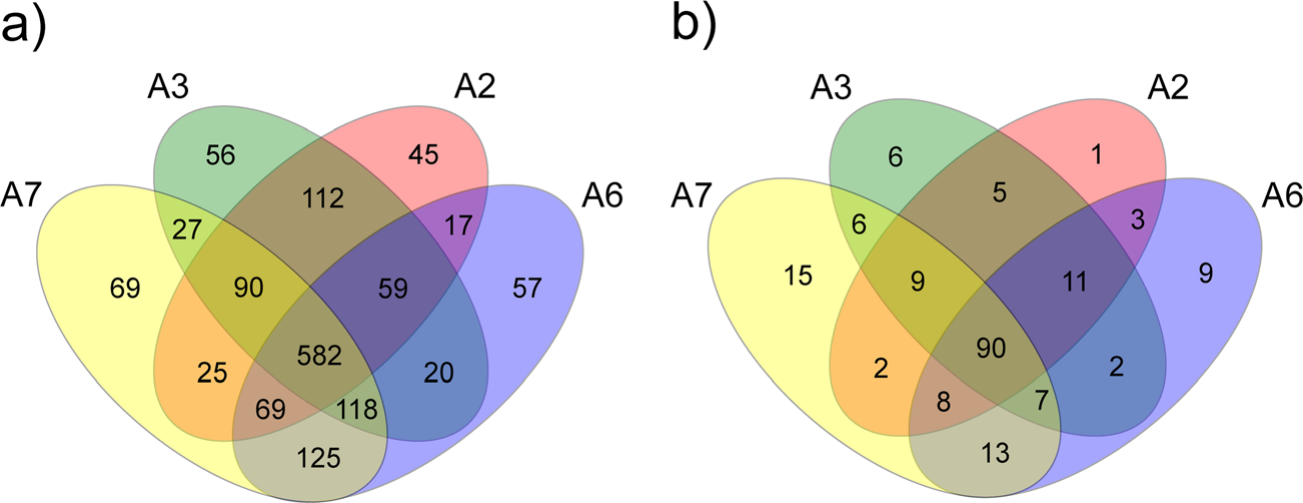
Venn diagram showing the shared and site-specific OTUs for nonculture-based bacterial (**a**) and fungal (**b**) communities at the four sampling locations (A2, A3, A6, A7)

The structure of the bacterial communities at the four sampling locations was significantly different according to PERMANOVA (*p* = 0.0097) and ANOSIM tests (*p* = 0.0092). However, both tests did not detect significant differences in the bacterial community structure between locations when they were pairwise compared (*p* > 0.33). The UPGMA dendrogram clustered the four locations in two different groups, one of them comprising A2 and A3 and the second one consisting of A6 and A7 (Fig. 4a). Approximately unbiased (AU) values of all clusters were >97%, i.e., the clustering was significant. These data were supported by the Venn diagram, which showed that the number of exclusive OTUs shared between A2 and A3 (112) and A6 and A7 (125) was much higher than that of any of the other pairwise sample comparisons (Fig. 5a). It is worth noting that only 582 OTUs (from a total of 1466) were found at all four sampling locations.

The bacterial groups responsible for the differences in the bacterial community structure were analyzed in detail. Although the proportion of the phylum Actinobacteria was constant at the four sampling locations (no significant changes were detected), its subclass *Rubrobacteridae* predominated at A6 (Table S2), and four of the six OTUs (classified as *Gaiellales* order) belonging to this group were significantly more abundant at A6 or A7 (Table S3). The subclass *Actinobacteridae* dominated A6 and A7; however, the largest OTU (OTU 2, see Table S3), classified as *Actinomycetales* order (*Actinobacteridae* class), was present at a significantly higher relative abundance at A2. On the other hand, the proportion of the subclass *Acidimicrobidae* was higher at A6 and A7 than at A2 and A3 (Tables S2 and S3). Within the phylum Proteobacteria, *Alphaproteobacteria* were more abundant at A6 and A7 (the analysis of major OTUs determined that, within classes, *Rhizobiales* predominated at A6 and A7), while *Deltaproteobacteria* (with the OTUs of this class belonging to the order *Syntrophobacterales*, Table S3) dominated at A2 and A3 (Table S2). The proportion of the phylum Acidobacteria was significantly higher at A2 and A3 than at A6 and A7. Only subgroups 6 and 4 of Acidobacteria significantly varied over the four sampling locations, predominating at A2 and A3 (Tables S2 and S3). The phyla Planctomycetes and Nitrospirae were most abundant at A3 and A2, respectively.

#### Fungal Communities

A total of 499,082 valid ITS1 region sequences, with an average read length of 140 bp, were obtained from the eight DNA extracts (two replicates per site). The average number of reads per replicate was 62,385 ± 27,685. The total sequence number represented a total of 188 different OTUs at 97% sequence identity, all of which were classified as fungi using a 50% threshold. The rarefaction curves showed that sequencing work was not fully exhaustive for any of the replicates per sampling location (Fig. S4). Taxonomic assignment analysis enabled the classification of ca. 86% sequences at the phylum level. The fungal diversity at the four sampling locations was distributed among five different phyla, with a dominance of Ascomycota (with the number of classified sequences in this phylum ranging from 46.6 to 87.4%), Basidiomycota (4.9–35.5%), and Zygomycota (0.1–9.3%) (Fig. 3b). Only ca. 63% of sequences could be classified at the class level, with *Pezizomycetes*, *Dothideomycetes*, and *Sordariomycetes* the predominant classes among Ascomycota; *Agaricomycetes* among Basidiomycota; and Incertidae sedis 10 (*Mortierellales* order) among Zygomycota (Fig. S3b).

The analysis of alpha diversity parameters showed that the fungal communities at the four sampling locations did not significantly vary (*p* > 0.05) in terms of richness, richness estimator ACE, Shannon diversity, or evenness (Table 2). However, ANOSIM (*p* = 0.0095) and PERMANOVA (*p* = 0.0108) analyses demonstrated significant differences of the fungal community structure between the four locations. The UPGMA dendrogram grouped the samples in two different clusters (Fig. 4b). A2 and A3 were grouped together with AU values ≥95. A6 and A7 comprised another group; however, due to an AU value of 76, this cluster was not significant (Fig. 4b). The Venn diagram demonstrated that 90 of the 188 fungal OTUs were shared between the four sampling locations (Fig. 5b).

Ascomycota were predominant at A3. Within this phylum, *Pezizales* dominated at A6 and A7, while *Pleosporales* prevailed at A2 and A3 (Table S4). The major OTU in the fungal library (OTU 4) belonged to the class *Pleosporales* and its relative abundance was significantly highest at A2 (Table S5). The phylum Basidiomycota was most abundant at A6; the order *Sebacinales* predominated at A6 and A7 (Table S4). No significant differences between the four sampling locations were found for the phyla Glomeromycota and Zygomycota.

### Community Composition and Structure of Culturable Heterotrophic Bacteria

From the four sampling locations, a total of 185 isolates (44, 46, 50, and 45 isolates from sites A2, A3, A6, and A7, respectively) were successfully identified according to their 16S rRNA sequences, which grouped into 27 different OTUs at 97% identity (Table S6). Approximately 82% of the strains shared ≥98.5% sequence identity with the closest known species in the EzTaxon Database, while ca. 17% of the isolates (sharing identities <98.5%) could constitute potential novel species.

Remarkably, in contrast to the nonculture-based approach, 90–100% of the culturable soil bacteria at each sampling location could be assigned to Gram-positive bacteria. The bacterial community was dominated by Actinobacteria (with relative abundances ranging from 64.44 to 93.18%), although isolates belonging to the phyla Proteobacteria and Firmicutes were also detected, especially at A6 and A7 (Fig. 3a). *Actinomycetales* was the predominant order. The culturable bacterial diversity clearly differed from that of the nonculture-based (Illumina) analysis (Fig. 3a). In fact, only one OTU of the top 32 most abundant nonculture-based bacterial OTUs (Table S3) was found among the isolates. The nonculture-based OTU 008 (Table S3) (classified as belonging to *Alphaproteobacteria*, *Rhizobiales* order) was phylogenetically related to the culturable OTU 015 (with identity values >97%) and consisted of two isolates belonging to the genus *Bosea* (*Rhizobiales*) (Table S6). OTUs of Actinobacteria dominated both in culture-dependent and culture-independent approaches; however, not one single strain belonging to the culturable actinobacterial OTUs could be assigned to the top 32 most abundant nonculture-based OTUs. Interestingly, one of the culturable isolates could be assigned to the bacterial candidate phylum TM7 (*Saccharibacteria*; superphylum *Patescibacteria*); no culturable representatives of this superphylum have been described so far.

The UPGMA dendrogram based on the distribution of the 27 culturable OTUs demonstrated that the culturable bacterial communities at A2 and A3 were more similar than those at A6 and A7 (Fig. S5).

## Discussion

In this study, we describe microbial communities in Archaic soil samples collected at four sampling locations situated at different places of a residential building, in order to answer the question if human consumption habits left traces on microbiota. Nonculture-based and culture-based approaches were applied to examine bacterial and fungal communities in terms of abundance, activity (functionality), diversity, and community structure.

Overall, all results obtained in this study demonstrated that soil microbial communities at sampling locations A2 and A3 (collected above the fireplace in the annex) clearly differed from those at sampling locations A6 and A7 (ritual deposit layers, filled with feasting rubbish after a festivity-related event) in the main room. Microbial characteristics at A2 and A3 were quite similar, while those at A6 and A7 showed some degree of dissimilarity, which can be attributed to different stages of deposition.

Soils were characterized by significantly higher contents of SOM, TOC, and N at A6 and A7 than at A2 and A3, and this was reflected by PLFA-derived microbial biomass amounts. However, total microbial and bacterial biomass was significantly highest at A7. This fact could be related to the nature of the organic material present at this sampling location. Despite comparable nutrient contents at A6 and A7, organic compounds at A7 were probably more accessible to microorganisms, which in turn resulted in a higher amount of biomass. This was corroborated by physiological profiles (CLPP) that demonstrated different patterns of microbial carbohydrate utilization at A6 and A7. CLPP analysis also showed that microorganisms at A2 and A3 were better adapted to the oxidation of carboxylic and acetic acids and showed a different pattern of polymer utilization. This is an indication of the presence of different substrates due to combustion processes at A2 and A3. The analysis of enzyme activities of culturable bacterial isolates highlighted that cellulose activity was only present among isolates of A6 and A7, which could point to the presence of plant materials in the past at these sampling locations.

Bacterial communities in terms of diversity differed. This could be related to site-specific bacterial abundance and/or characteristics since also richness and the abundance-based diversity index ACE were significantly higher at A7 than at the three other sampling locations. On the other hand, it was remarkable that fungal communities seemed to be less affected by site-specific properties since neither PLFA-derived fungal biomass nor Illumina-based abundance or diversity differed between the four sampling locations. However, fungal community structure analysis clearly showed similar fungal communities at A2 and A3, differing from those at A6 and A7. The same result was obtained for the analysis of the bacterial community structure, both with culture-based and nonculture-based techniques.

In terms of taxonomy, soil bacterial communities at the four sampling locations, independent whether culture-based or nonculture-based approaches were applied, were dominated by Gram-positive bacteria belonging to Actinobacteria. This bacterial group exhibits a wide range of morphologies, lifestyles, and physiological and metabolic properties; members of some orders are spore-formers and thus able to withstand unfavorable conditions [45]. They are widely distributed in soil ecosystems, where they play a crucial role in the recycling of biomaterials by decomposition and humus formation [46]. The divergence of Actinobacteria from other bacteria is ancient [47]. Actinobacteria are *K* strategists that tend to be more successful in resource-limited habitats and are usually more stable and permanent members of the microbial community [48]. Although Actinobacteria dominated at the four sampling locations, there were differences in the relative abundance of subclasses of this phylum, for example, *Rubrobacteridae* and *Actinobacteridae* were more abundant at A6 and A7 than at A2 and A3.

Fungal diversity was dominated by *Pezizomycetes* and *Dothideomycetes* classes (Ascomycetes), which have been often described in xeric environments [49, 50], an environmental property than can also be ascribed to the four sampling locations of our study. The high abundance of *Pezizomycetes* and *Dothideomycetes* was also noted in a study on the microbial diversity of ancient wall paintings [51].

For both bacteria and fungi, it was remarkable that a high proportion of sequences could not be classified at a taxonomic level. There are two possible explanations: either the sequence size may have been too short for accurate classification, or the database may not be complete enough to include all taxonomic information for each phylum and, as a result, some comparative elements were missing for the classification of the complete microbial diversity [52]. The first explanation could justify the high proportion of unclassified fungal sequences (average read length of 140 bp); however, in the case of bacteria, the second reason is more plausible as the bacterial sequences length was larger than 400 bp due to the selection of the primer pair 27F and 519R. Therefore, the high level of unclassified bacterial sequences could be an indication of a high degree of undescribed diversity at the studied sampling locations. This was also corroborated by results obtained from the culture-dependent study, which, although not exhaustive, resulted in a high proportion of potentially novel species. In this context, it is also worth noting that few culturable isolates belonged to the most representative nonculture-based OTUs. This could be attributed to the presence of a high proportion of nonculturable bacteria or of bacteria in the viable but nonculturable (VBNC) state.

In contrast to PLFA-derived (living) bacterial biomass, numbers of culturable bacteria were significantly higher at A2 and A3 than at A6 and A7. This can be explained by the fact that culturable bacteria could be assigned almost exclusively to Gram-positive bacteria, especially Actinobacteria, which were found among nonculture-based communities with similar abundance at all four sampling locations. In addition, *Micrococcales* and *Bacillales*, the most abundant orders among Actinobacteria, survive extreme conditions, such as prevailing on fireplaces, due to sporulation [53]. The predominance of Actinobacteria among culturable bacteria was also detected in soil samples from pottery vessels at an archaeological site in South Italy [54]. Similarly, Actinobacteria dominated among culturable and nonculturable bacterial communities in exfoliated sandstone of a medieval castle [55], which was explained by the particular capacity of Actinobacteria to survive adverse environmental conditions, such as low water availability and elevated temperatures [56].

Culture-based data demonstrated the dominance of Gram-positive bacteria (91–100% at all four sites), while nonculture-based data at the phylum level showed an almost equal distribution between Gram-positive and Gram-negative bacteria. This discrepancy can be related to a number of facts. DNA extraction from Gram-positive bacteria may not be as effective as from Gram-negative bacteria since Gram-positive bacteria are harder to lyse because of their cell wall structure [57]. Culture-independent analysis includes sequences from microorganisms that are not yet cultured or are difficult to culture, and only a small fraction of the microbial community is culturable. The low recovery of viable cells from ancient habitats can be explained by a high amount of cells in the VBNC state [58]. Microbial long-term survival has often been questioned; however, there are several reports on the successful isolation/revival of microorganisms from archaeological sites. For example, active microorganisms were found in soils in prehistoric settlements and their different characteristics were attributed to varying human impacts at archaeological sites [3]. Sporogenic bacteria and fungi were revived from soil samples from a stratified deposit under a house buried 4000 years ago at a Bronze Age archaeological site in Iran [59]. Viable microorganisms were isolated from soil samples from pottery vessels at two archaeological sites in South Italy (dated 1053 AD) and Belize (dated 700–900 AD) [54].

Considerable microbial abundance and diversity was found at all four Archaic soil sampling locations, and our study clearly demonstrates differences in microbial community structure and diversity. These differences can be related to various consumer are(n)as or consumptionscapes. From an archaeological viewpoint, the discrepancy between microbial characteristics at A6 and A7 needs explanation, since samples from both sampling locations were collected from the filling of the deposit in the main room of the native building. Sherd-links of a matt-painted jug and a storage vessel, both from the lower layer (A7) and the upper layer (A6), indicate that both layers are the result of an act of ritual deposition [18]. This macroarchaeological finding does not indicate that both layers in the deposit arose from two different activities, which, however, was evidenced from microarchaeological findings. The different microbial characteristics at A6 und A7 can be attributed to different microbial responses to particular consumer habits. Thus, different habits of consumption or consumptionscapes resulted in divergent compositions of rubbish in the deposit and consequently left different microbial traces.

This finding has an influence on the reconstruction of events that led to the digging and filling of the deposit in the main room of the native dwelling. After the festivity on the occasion of the ritual abandonment of the native dwelling, “feasting garbage” from two different consumeristic zones was used to fill the deposit. First, material present in the lower layer (A7) was used for filling, then this layer was sealed with a layer of loam and pebbles. In a third act, the feasting dump present in the upper layer (A6) was added. All this happened in the course of one and the same ritual act.

Our study is one of the first integrative reports of microbiology and archaeology to reconstruct human activities at an ancient settlement. It seems to be clear that the so-called ecofacts are not unmodified pieces of evidence, influenced by nature rather than by humans [12, 13]. On the contrary, as shown with soil samples from two deposit layers (A6 and A7), “ecofacts” were moved from the are(n)a of primary activity to the area of deposition, selected by people, ritually discarded and intentionally modified, which finally resulted in the development of different microbiota. Therefore, “ecofacts” are, just like artifacts, “the result of human activities” [14] and important indicators of consumeristic behaviors. Clearly, also the microbial residues in the archaeological evidence represent such consumer-produced “ecofacts” (therefore conceptualized as “vivifacts”) [60]. So far, the archaeological benefit of soil microorganisms from ancient cultural layers was mainly restricted to the reconstruction of climates and environmental properties in the past ([4] and references therein). Our study reveals further potential of microbiology for archaeology, which still has to be discovered in full [4]. In this respect, the traditional, artifact-oriented archaeology can interact with natural sciences to gain more information about consumer or ritual behaviors on Archaic Monte Iato and elsewhere in the distant past [19].

## Conclusion

Here, we report the first examination of soil bacterial and fungal communities in the anthropogenic layers of a residential building of the 6th cent. BC at Monte Iato (Sicily, Italy). We investigated the upper and the lower infill of a ritual deposit (food waste disposal) in the main room (sampling locations A6, A7) as well as the strata above the fireplace in the annex (sampling locations A2, A3). Soil physicochemical and microbial properties (including biomass, activity, diversity, and community structure) above the fireplace (A2, A3) were clearly different from the properties in soils related to the ritual deposit (A6, A7). These differences demonstrate that anthropogenic consumption habits in the past left traces on microbiota. The use of microbial bioindication is a significant complementation of traditional archaeological methods.

## Electronic Supplementary Material

Below is the link to the electronic supplementary material.

ESM 1 (PDF 467 kb)
