# Supplementary material for: Microbiology Meets Archaeology: Soil Microbial Communities Reveal Different Human Activities at Archaic Monte Iato (Sixth Century BC)
Source: Microb Ecol. 2016 Dec 13;73(4):925–38. doi: 10.1007/s00248-016-0904-8 (PMC5382179; doi:10.1007/s00248-016-0904-8)

SUPPLEMENTARY MATERIAL

Microbiology Meets Archaeology: Soil Microbial Communities Reveal Different Human Activities at Archaic Monte Iato (6th Cent. BC)

Rosa Margesin*, José A. Siles, Tomas Cajthaml, Birgit Öhlinger, Erich Kistler

*Corresponding author: Rosa Margesin; e-mail: Rosa.Margesin@uibk.ac.at

Journal: Microbial Ecology

The supplementary material includes Tables S1-S6 and Figures S1-S5

Table S1 Relative oxidation rates of CLPP C-source families at the sampling locations A2, A3, A6 and A7. Values are expressed as AWCD. For each variable, data followed by different letters are significantly different ($p \leq 0.05$).

C-source family	A2	A3	A6	A7
Polymers	0.38 **a**	0.30 **a**	0.50 **a**	0.15 **a**
Carbohydrates	0.63 **a**	0.48 **a**	1.02 **ab**	1.58 **b**
Carboxylic and acetic acids	1.16 **ab**	1.63 **b**	0.14 **a**	0.52 **a**
Amino acids	0.66 **a**	0.61 **a**	0.50 **a**	0.23 **a**
Amines/acides	0.16 **a**	0.22 **a**	0.04 **a**	0.25 **a**

Table S2 Relative abundances (%) of most abundant non-culturable bacterial phyla and predominant subclasses of Actinobacteria, classes of Proteobacteria and subgroups of Acidobacteria at the four sampling locations. Data represent means. In each row, data followed by different letters are significantly different ($p \leq 0.05$).

**Taxa**	**A2**	**A3**	**A6**	**A7**
Actinobacteria	30.10 **a**	28.02 **a**	42.11 **a**	37.60 **a**
Rubrobacteridae	4.24 **a**	5.59 **a**	17.24 **b**	8.69 **a**
Actinobacteridae	4.77 **a**	7.25 **ab**	8.65 **bc**	10.35 **c**
Acidimicrobidae	0.39 **a**	0.32 **a**	1.12 **c**	0.69 **b**
Proteobacteria	10.97 **a**	13.71 **a**	12.03 **a**	16.36 **a**
Alphaproteobacteria	2.48 **a**	5.25 **ab**	8.56 **b**	10.26 **b**
Deltaproteobacteria	3.30 **bc**	4.49 **c**	1.35 **a**	2.10 **ab**
Gammaproteobacteria	3.15 **a**	2.30 **a**	0.94 **a**	1.67 **a**
Betaproteobacteria	1.93 **b**	1.57 **ab**	1.02 **a**	2.19 **b**
Acidobacteria	15.26 **b**	16.83 **b**	10.14 **a**	10.15 **a**
Gp 6	5.29 **bc**	7.00 **c**	3.59 **ab**	3.18 **a**
Gp 4	3.46 **a**	4.01 **a**	3.74 **a**	3.67 **a**
Gp 7	3.87 **b**	3.47 **b**	1.08 **a**	1.61 **a**
Gp 18	0.98 **a**	0.69 **a**	0.35 **a**	0.37 **a**
Gp 16	0.46 **a**	0.43 **a**	0.82 **a**	0.51 **a**
Bacteroidetes	2.11 **a**	1.74 **a**	6.66 **a**	6.38 **a**
Planctomycetes	5.50 **ab**	7.57 **b**	4.19 **ab**	2.87 **a**
Nitrospirae	2.78 **b**	2.42 **ab**	2.31 **a**	3.91 **a**
Others	6.49 **a**	4.86 **a**	5.51 **a**	5.90 **a**
Unclassified	26.79 **a**	24.85 **a**	17.04 **a**	16.83 **a**

Table S3 Identification and relative abundances of non-culturable bacterial OTUs with abundances >0.5% at the sampling locations A2, A3, A6 and A7. Data represent means. For each OTU, data followed by different letters are significantly different (p≤0.05).

**OTU No.**	**OTU ID**	**Phylum (Class)**	**Order**	**A2**	**A3**	**A6**	**A7**
1	OTU_002	Actinobacteria (*Actinobacteria*)	*Actinomycetales*	20.07 **b**	10.21 **a**	8.30 **a**	11.42 **ab**
2	OTU_005	Actinobacteria (*Actinobacteria*)	*Acidimicrobiales*	8.45 **ab**	4.90 **a**	21.25 **c**	15.40 **bc**
3	OTU_006	Aquificae (*Aquificae*)	*Aquificales*	20.31 **b**	26.38 **c**	1.07 **a**	2.23 **a**
4	OTU_011	Actinobacteria (*Thermoleophilia*)	*Thermoleophilaceae*	2.42 **a**	4.88 **a**	20.15 **b**	22.55 **b**
5	OTU_048	Acidobacteria (Gp4)	Gp4	9.97 **a**	13.91 **a**	15.19 **a**	10.93 **a**
6	OTU_003	Proteobacteria (*Alphaproteobacteria*)	*Rhodospirillales*	1.78 **a**	4.83 **a**	20.78 **a**	22.61 **a**
7	OTU_014	Actinobacteria (*Actinobacteria*)	*Actinomycetales*	17.33 **b**	16.22 **b**	5.78 **a**	10.66 **ab**
8	OTU_015	Actinobacteria (*Actinobacteria*)	*Gaiellales*	2.04 **a**	2.04 **a**	35.69 **c**	10.23 **b**
9	OTU_025	Acidobacteria (Gp6)	Gp6	15.24 **ab**	24.85 **b**	5.74 **a**	4.18 **a**
10	OTU_033	Nitrospirae (*Nitrospira*)	*Nitrospirales*	14.16 **a**	12.65 **a**	7.83 **a**	15.36 **a**
11	OTU_019	Actinobacteria (*Actinobacteria*)	*Acidimicrobiales*	13.46 **a**	12.66 **a**	10.90 **a**	12.98 **a**
12	OTU_017	Proteobacteria (*Deltaproteobacteria*)	*Syntrophobacterales*	14.86 **ab**	18.66 **b**	6.58 **a**	9.90 **ab**
13	OTU_008	Proteobacteria (*Alphaproteobacteria*)	*Rhizobiales*	3.42 **a**	9.54 **a**	13.07 **ab**	23.97 **b**
14	OTU_035	Aquificae (*Aquificae*)	*Aquificales*	26.22 **b**	23.26 **b**	0.25 **a**	0.27 **a**
15	OTU_021	Proteobacteria (*Deltaproteobacteria*)	*Syntrophobacterales*	18.40 **b**	27.20 **c**	0.77 **a**	3.63 **a**
16	OTU_128	Actinobacteria (*Actinobacteria*)	*Gaiellales*	2.53 **a**	6.30 **a**	33.25 **b**	7.92 **a**
17	OTU_026	Bacteroidetes (Bacteroidetes_incertae_sedis)	*Ohtaekwangia*	16.51 **b**	14.34 **ab**	5.44 **a**	13.71 **a**
18	OTU_010	Chloroflexi (*Anaerolineae*)	*Anaerolineales*	1.85 **a**	1.20 **a**	31.64 **c**	15.31 **b**
19	OTU_080	Acidobacteria (Gp4)	Gp4	19.42 **b**	10.56 **ab**	10.04 **a**	9.99 **a**
20	OTU_190	Actinobacteria (*Actinobacteria*)	*Actinomycetales*	1.32 **a**	0.94 **a**	23.66 **a**	24.08 **a**
21	OTU_057	Acidobacteria (Gp7)	Gp7	20.18 **b**	21.16 **b**	2.84 **a**	5.83 **a**
22	OTU_050	Acidobacteria (Gp6)	Gp6	17.45 **b**	21.57 **b**	3.38 **a**	7.60 **a**
23	OTU_038	Chloroflexi (*Thermoflexia*)	*Thermoflexales*	11.94 **a**	24.29 **b**	4.42 **a**	9.35 **a**
24	OTU_175	Nitrospirae (*Nitrospira*)	*Nitrospirales*	14.05 **a**	5.93 **a**	10.70 **a**	19.32 **a**
25	OTU_023	Actinobacteria (*Actinobacteria*)	*Actinomycetales*	1.82 **a**	11.64 **a**	9.44 **a**	27.10 **a**
26	OTU_059	Actinobacteria (*Actinobacteria*)	*Gaiellales*	3.72 **a**	6.37 **a**	31.17 **b**	8.74 **a**
27	OTU_207	Actinobacteria (*Actinobacteria*)	*Gaiellales*	26.70 **b**	17.63 **ab**	1.30 **a**	4.37 **a**
28	OTU_249	Gemmatimonadetes (*Gemmatimonadetes*)	*Gemmatimonadales*	29.04 **a**	9.74 **a**	1.60 **a**	9.62 **a**
29	OTU_027	Bacteroidetes (Bacteroidetes_incertae_sedis)	*Ohtaekwangia*	0.18 **a**	0.12 **a**	1.11 **a**	48.60 **a**
30	OTU_118	Actinobacteria (*Actinobacteria*)	*Gaiellales*	13.95 **b**	14.78 **b**	4.49 **a**	16.78 **b**
31	OTU_121	Actinobacteria (*Actinobacteria*)	*Gaiellales*	1.08 **a**	1.74 **a**	34.09 **b**	13.09 **a**
32	OTU_072	Firmicutes (*Clostridia*)	*Thermoanaerobacterales*	6.82 **a**	22.79 **c**	5.22 **a**	15.17 **b**

Table S4 Relative abundances (%) of non-culturable fungal phyla and the most abundant orders of Ascomycota, Basidiomycota and Zygomycota at the sampling locations A2, A3, A6 and A7. Data represent means. In each row, data followed by different letters are significantly different ($p \leq 0.05$).

**Taxa**	**A2**	**A3**	**A6**	**A7**
Ascomycota	79.59 **ab**	87.84 **b**	46.62 **a**	64.33 **ab**
Pezizales	0.69 **a**	0.37 **a**	35.05 **b**	45.52 **c**
Pleosporales	49.91 **b**	47.03 **b**	0.38 **a**	0.32 **a**
Hypocreales	19.40 **a**	23.80 **a**	3.78 **a**	9.04 **a**
Helotiales	0.51 **a**	8.81 **a**	0.04 **a**	1.63 **a**
Eurotiales	0.49 **a**	0.28 **a**	0.62 **a**	3.81 **b**
Basidiomycota	5.63 **a**	4.85 **a**	35.46 **b**	3.70 **a**
Sebacinales	0.02 **a**	0.03 **a**	2.50 **b**	2.43 **b**
Agaricales	0.60 **a**	1.15 **a**	0.59 **a**	0.56 **a**
Zygomycota	9.31 **a**	4.06 **a**	0.13 **a**	0.81 **a**
Mortierellales	9.31 **a**	4.06 **a**	0.13 **a**	0.81 **a**
Glomeromycota	0.02 **a**	0.02 **a**	2.60 **a**	0.66 **a**
Chytridiomycota	0.01 **a**	0.02 **a**	0.01 **a**	0.00 **a**
Unclassified	5.44 **a**	3.22 **a**	15.17 **a**	30.51 **a**

Table S5 Identification and relative abundances (%) of non-culturable fungal OTUs with abundance >0.5% at the sampling locations A2, A3, A6 and A7. Data represent means. For each OTU, data followed by different letters are significantly different ($p \leq 0.05$).

OTU No.	OTU ID	Phylum (Class)	Order	A2	A3	A6	A7
1	OTU_004	Ascomycota (*Dothideomycetes*)	*Pleosporales*	31.39 **b**	18.35 **ab**	0.12 **a**	0.14 **a**
2	OTU_002	Ascomycota (*Pezizomycetes*)	*Pezizales*	0.30 **a**	0.42 **a**	10.45 **a**	38.82 **b**
3	OTU_003	Ascomycota (*Sordariomycetes*)	*Hypocreales*	15.10 **a**	23.47 **a**	2.60 **a**	8.83 **a**
4	OTU_007	Basidiomycota (*Tremellomycetes*)	*Tremellales*	0.31 **a**	0.27 **a**	49.17 **b**	0.26 **a**
5	OTU_006	Ascomycota (*Dothideomycetes*)	*Pleosporales*	0.19 **a**	49.48 **a**	0.12 **a**	0.21 **a**
6	OTU_005	Basidiomycota (*Agaricomycetes*)	*Polyporales*	0.19 **a**	0.32 **a**	4.02 **a**	45.47 **b**
7	OTU_008	Ascomycota (Ascomycota unidentified)	Ascomycota unidentified 1	0.20 **a**	0.28 **a**	49.20 **a**	0.31 **a**
8	OTU_010	Basidiomycota (*Agaricomycetes*)	*Agaricales*	0.19 **a**	0.20 **a**	0.27 **a**	49.34 **a**
9	OTU_011	Ascomycota (*Dothideomycetes*)	*Pleosporales*	28.30 **a**	20.97 **a**	0.37 **a**	0.37 **a**
10	OTU_014	Ascomycota (*Pezizomycetes*)	*Pezizales*	0.13 **a**	0.14 **a**	47.28 **a**	2.46 **a**
12	OTU_026	Basidiomycota (*Agaricomycetes*)	*Sebacinales*	0.14 **a**	0.11 **a**	35.84 **a**	13.92 **a**
13	OTU_012	Ascomycota (*Leotiomycetes*)	*Helotiales*	0.31 **a**	49.32 **b**	0.19 **a**	0.18 **a**
14	OTU_013	Ascomycota (*Pezizomycetes*)	*Pezizales*	0.31 **a**	0.18 **a**	48.88 **a**	0.63 **a**
15	OTU_016	Basidiomycota (*Agaricomycetes)*	*Cantharellales*	0.11 **a**	0.23 **a**	49.43 **a**	0.23 **a**
16	OTU_015	Ascomycota (*Eurotiomycetes*)	*Eurotiales*	0.17 **a**	0.25 **a**	5.75 **a**	43.**83 b**
17	OTU_023	Basidiomycota (*Agaricomycetes*)	*Agaricales*	49.34 **a**	0.16 **a**	0.28 **a**	0.23 **a**
18	OTU_019	Zygomycota (Incertae sedis 10)	*Mortierellales*	48.06 **a**	1.06 **a**	0.29 **a**	0.59 **a**
19	OTU_020	Zygomycota (Incertae sedis 10)	*Mortierellales*	35.48 **a**	11.37 **a**	0.32 **a**	2.84 **a**
20	OTU_017	Ascomycota (*Leotiomycetes*)	*Helotiales*	0.12 **a**	0.22 **a**	0.36 **a**	49.30 **b**
21	OTU_021	Glomeromycota (Glomeromycota_unidentified)	Glomeromycota unidentified 1	0.21 **a**	0.23 **a**	39.05 **b**	10.51 **a**
22	OTU_027	Fungi_unidentified (Fungi unidentified 1)	Fungi unidentified 1	49.13 **a**	0.24 **a**	0.46 **a**	0.17 **a**
23	OTU_022	Ascomycota (*Sordariomycetes*)	*Hypocreales*	6.47**a**	28.34 **b**	5.85 **a**	9.34 **a**
24	OTU_060	Ascomycota (*Dothideomycetes*)	*Pleosporales*	36.25 **a**	13.03 **a**	0.39 **a**	0.33 **a**
25	OTU_044	Basidiomycota (*Tremellomycetes*)	*Cystofilobasidiales*	0.10 **a**	0.00 **a**	49.70 **a**	0.20 **a**
26	OTU_031	Ascomycota (*Pezizomycetes*)	*Pezizales*	0.14 **a**	0.24 **a**	49.29 **a**	0.34 **a**
27	OTU_033	Ascomycota (*Leotiomycetes*)	*Helotiales*	0.14 **a**	0.32 **a**	0.39 **a**	49.16 **b**
28	OTU_036	Ascomycota (*Sordariomycetes*)	*Hypocreales*	42.25 **a**	7.03 **a**	0.40 **a**	0.32 **a**
29	OTU_049	Ascomycota (Ascomycota unidentified)	Ascomycota unidentified 1	40.47 **a**	8.84 **a**	0.22 **a**	0.47 **a**
30	OTU_028	Zygomycota (Incertae sedis 10)	*Mortierellales*	31.98 **b**	10.57 **a**	0.11 **a**	7.34 **a**
31	OTU_029	Ascomycota (*Sordariomycetes*)	*Hypocreales*	32.83 **a**	9.36 **a**	1.69 **a**	6.12 **a**
32	OTU_037	Ascomycota (*Dothideomycetes*)	*Pleosporales*	44.79 **a**	4.79 **a**	0.26 **a**	0.15 **a**
33	OTU_053	Ascomycota (*Sordariomycetes*)	*Hypocreales*	0.91 **a**	45.97 **b**	0.84 **a**	2.28 **a**
34	OTU_034	Ascomycota (Ascomycota unidentified)	Ascomycota unidentified 1	29.14 **b**	13.56 **a**	6.30 **a**	0.99 **a**
35	OTU_032	Ascomycota (*Dothideomycetes*)	Dothideomycetes unidentified	48.89 **a**	0.40 **a**	0.36 **a**	0.36 **a**
36	OTU_038	Basidiomycota (*Agaricomycetes*)	*Sebacinales*	0.19 **a**	0.14 **a**	6.60 **a**	43.07 **a**
37	OTU_030	Zygomycota (Incertae sedis 10)	*Mortierellales*	0.43 **a**	47.42 **b**	1.00 **a**	1.15 **a**
38	OTU_052	Ascomycota (Incertae sedis 14)	Incertae sedis 15	0.29 **a**	24.35 **a**	19.23 **a**	6.12 **a**

Table S6 Identification of the culturable bacterial OTUs and their distribution at the sites A2, A3, A6, and A7.

OTU no.	OTU Id	Isolate Id	GenBank access. no.	Closest relative Eztaxon match (GenBank accession no.), % similarity	Phylum (order)	Number of isolates per site			
						A2	A3	A6	A7
1	OTU_01	A3.51	KX028737	*Arthrobacter oxydans* DSM 20119(T) (X83408), 100.00	Actinobacteria (*Actinomycetales*)	35	34	6	10
2	OTU_02	A7.30	KX028738	*Arthrobacter nicotinovorans* DSM 420(T) (X80743), 97.51	Actinobacteria (*Actinomycetales*)	0	4	8	2
3	OTU_03	A7.07	KX028739	*Nocardioides albus* KCTC 9186(T) (AF004988), 99.51	Actinobacteria (*Propionibacteriales*)	3	1	8	2
4	OTU_04	A7.09	KX028740	*Bacillus simplex* NBRC 15720(T) (AB363738), 100.00	Firmicutes (*Bacillales*)	0	2	1	9
5	OTU_05	A7.49	KX028741	*Agromyces humatus* CD5(T) (AY618216), 97.64	Actinobacteria (*Actinomycetales*)	1	2	3	4
6	OTU_06	A6.48	KX028742	*Paenibacillus pectinilyticus* RCB-08(T) (EU391157), 99.14	Firmicutes (*Bacillales*)	1	0	6	2
7	OTU_07	A7.50	KX028743	*Promicromonospora xylanilytica* YIM 61515(T) (FJ214352), 99.50	Actinobacteria (*Actinomycetales*)	0	0	3	2
8	OTU_08	A6.06	KX028744	*Variovorax soli* GH 9-3(T) (DQ432053), 99.18	Proteobacteria (*Burkholderiales*)	0	0	4	0
9	OTU_09	A7.29	KX028745	*Pseudomonas lini* CFBP 5737(T) (AY035996), 99.00	Proteobacteria (*Pseudomonadales*)	0	0	1	2
10	OTU_10	A7.36	KX028746	*Agromyces neolithicus* 23-23(T) (AY507128), 97.99	Actinobacteria (*Actinomycetales*)	0	0	1	2
11	OTU_11	A7.45	KX028747	*Streptomyces sioyaensis* NRRL B-5408(T) (DQ026654), 99.69	Actinobacteria (*Streptomycetales*)	0	0	2	1
12	OTU_12	A7.51	KX028748	*Microbacterium flavescens* DSM 20643(T) (Y17232), 98.31	Actinobacteria (*Actinomycetales*)	0	0	0	3
13	OTU_13	A6.41	KX028749	*Streptomyces puniceus* NRRL ISP-5058(T) (JOAD01000551), 98.30	Actinobacteria (*Streptomycetales*)	0	0	2	0
14	OTU_14	A7.33	KX028750	*Agromyces ramosus* DSM 43045(T) (X77447), 97.76	Actinobacteria (*Actinomycetales*)	0	0	1	1
15	OTU_15	A2.05	KX028751	*Bosea eneae* 34614(T) (AF288300), 99.52	Proteobacteria (*Rhizobiales*)	2	0	0	0
16	OTU_16	A7.08	KX028752	*Paenibacillus endophyticus* PECAE04(T) (KC447384), 99.08	Firmicutes (*Bacillales*)	0	0	1	1
17	OTU_17	A6.22	KX028753	*Paenibacillus algorifonticola* XJ259(T) (GQ383922), 99.68	Firmicutes (*Bacillales*)	0	0	2	0
18	OTU_18	A3.09	KX028754	*Arthrobacter oryzae* KV-651(T) (AB279889), 97.90	Actinobacteria (*Actinomycetales*)	0	1	0	0
19	OTU_19	A3.30	KX028755	*Fictibacillus phosphorivorans* Ca7T(T) (JX258924), 99.48	Firmicutes (*Bacillales*)	0	1	0	0
20	OTU_20	A3.16	KX028756	*Bacillus ginsengisoli* DCY53(T) (HQ224517), 98.48	Firmicutes (*Bacillales*)	0	1	0	0
21	OTU_21	A7.32	KX028757	*Phyllobacterium brassicacearum* STM 196(T) (AY785319), 97.27	Proteobacteria (*Rhizobiales*)	0	0	0	1
22	OTU_22	A7.48	KX028758	*Paenibacillus pectinilyticus* RCB-08(T) (EU391157), 99.14	Firmicutes (*Bacillales*)	0	0	0	1
23	OTU_23	A7.24	KX028759	*Ensifer garamanticus* ORS 1400(T) (AY500255), 99.32	Proteobacteria (*Rhizobiales*)	0	0	0	1
24	OTU_24	A2.46	KX028760	*Aeromicrobium panaciterrae* Gsoil 161(T) (AB245387), 99.74	Actinobacteria (*Propionibacteriales*)	1	0	0	0
25	OTU_25	A2.38	KX028761	Candidatus *Saccharimonas aalborgensis* (CP005957), 91.24	Saccharibacteria (*Saccharimonas*)	1	0	0	0
26	OTU_26	A7.41	KX028762	*Paenibacillus alginolyticus* DSM5050T(T) (D78465), 99.68	Firmicutes (*Bacillales*)	0	0	0	1
27	OTU_27	A6.23	KX028763	*Microbacterium flavescens* DSM 20643(T) (Y17232), 94.70	Actinobacteria (*Actinomycetales*)	0	0	1	0

Fig. S1 Cult precinct south of Peristyle House I, 500–460 B.C. (**A**, Aphrodite-Temple; **B**, Late Archaic House; **C**, native building (Institute of Archaeologies, University of Innsbruck).

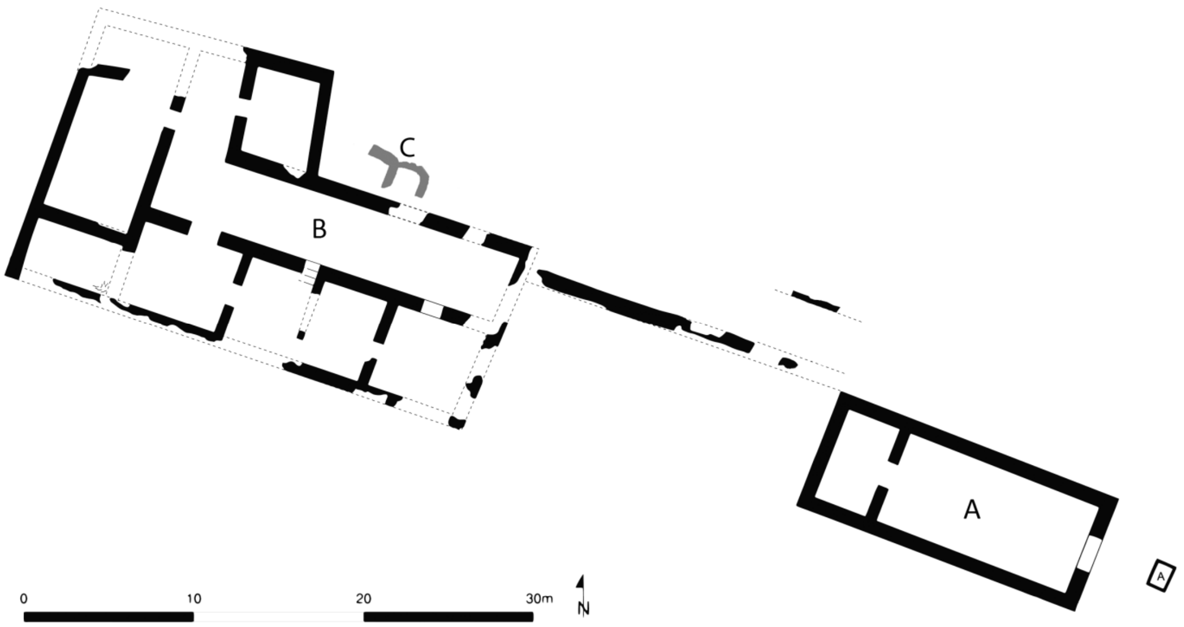

Fig. S2 Rarefaction curves for non-culturable bacterial communities found at the sampling locations A2, A3, A6 and A7.

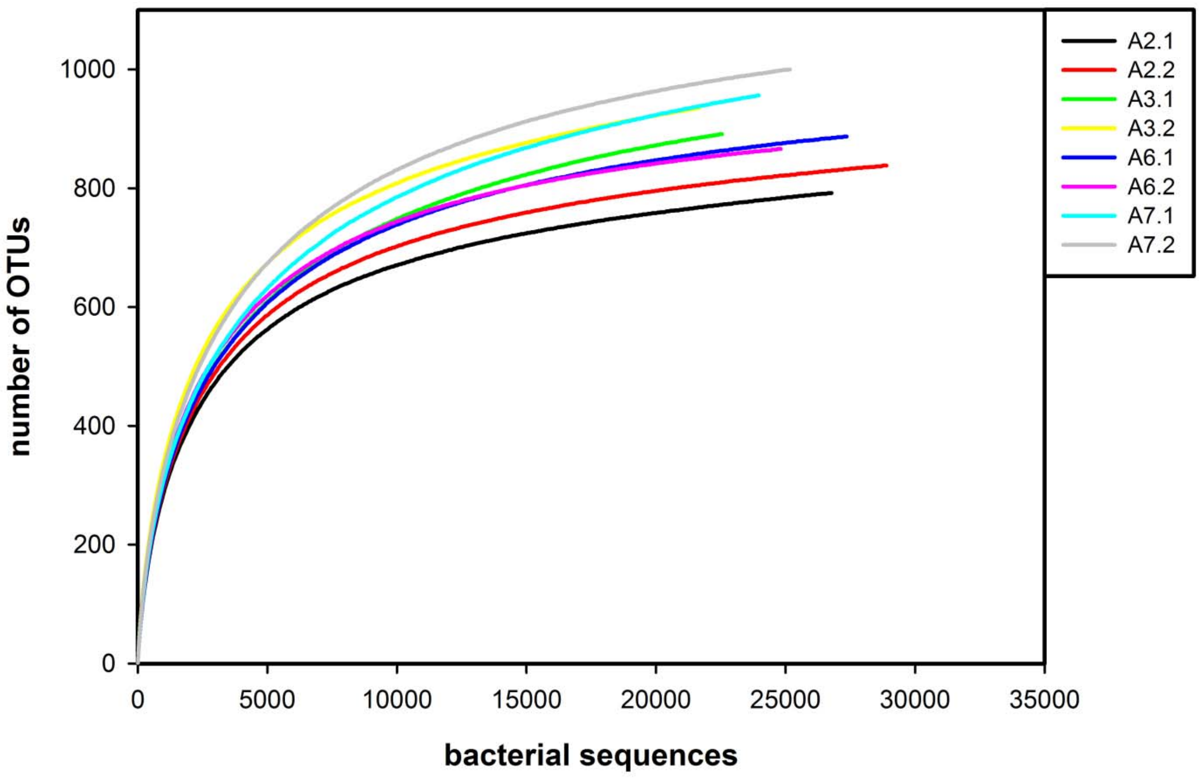

Fig. S3 Relative abundance (%) of non-culturable bacterial (**a**) and fungal (**b**) classes found at the sampling locations A2, A3, A6 and A7.

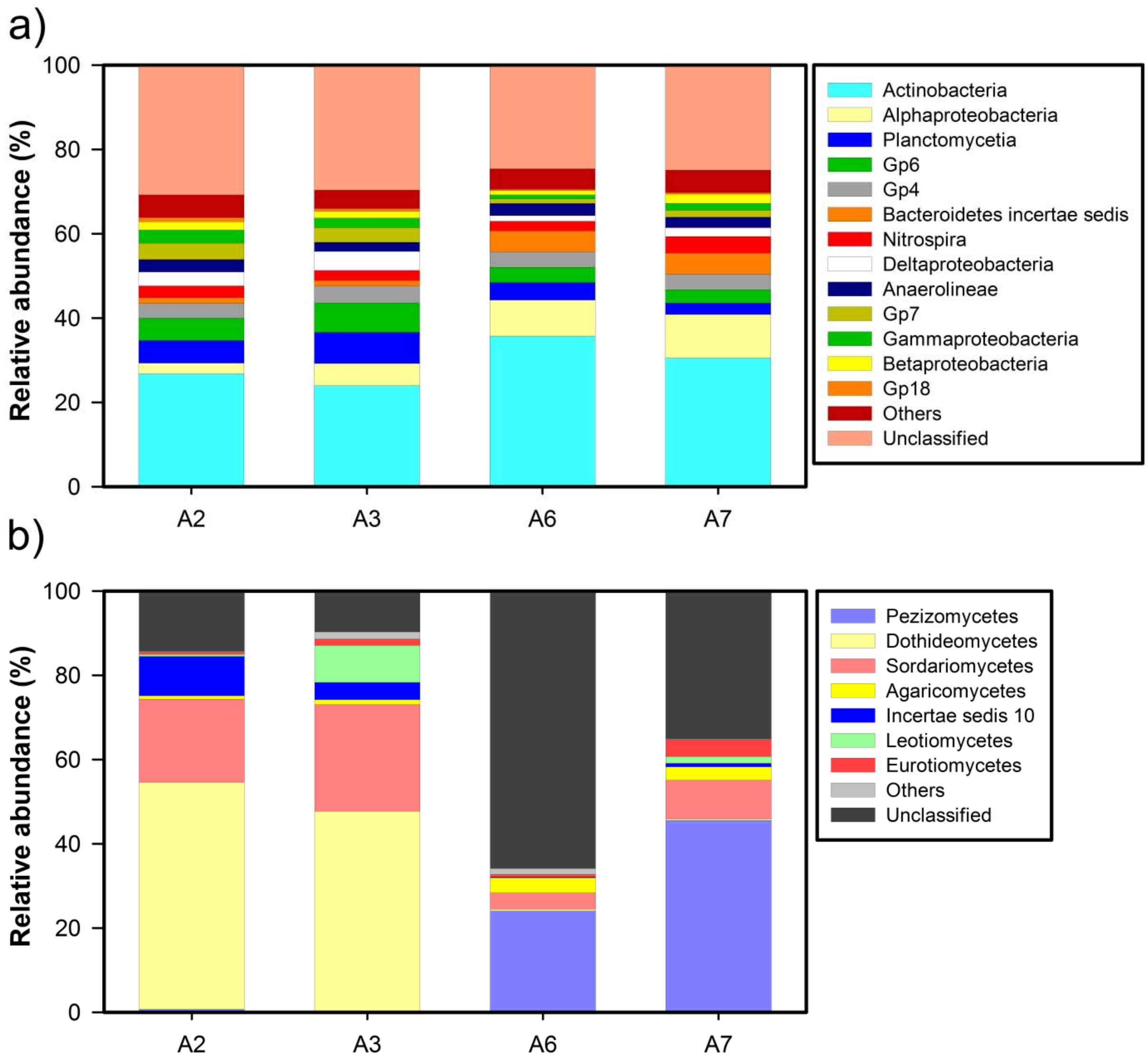

Fig. S4 Rarefaction curves for non-culturable fungal communities found at the sampling locations A2, A3, A6 and A7.

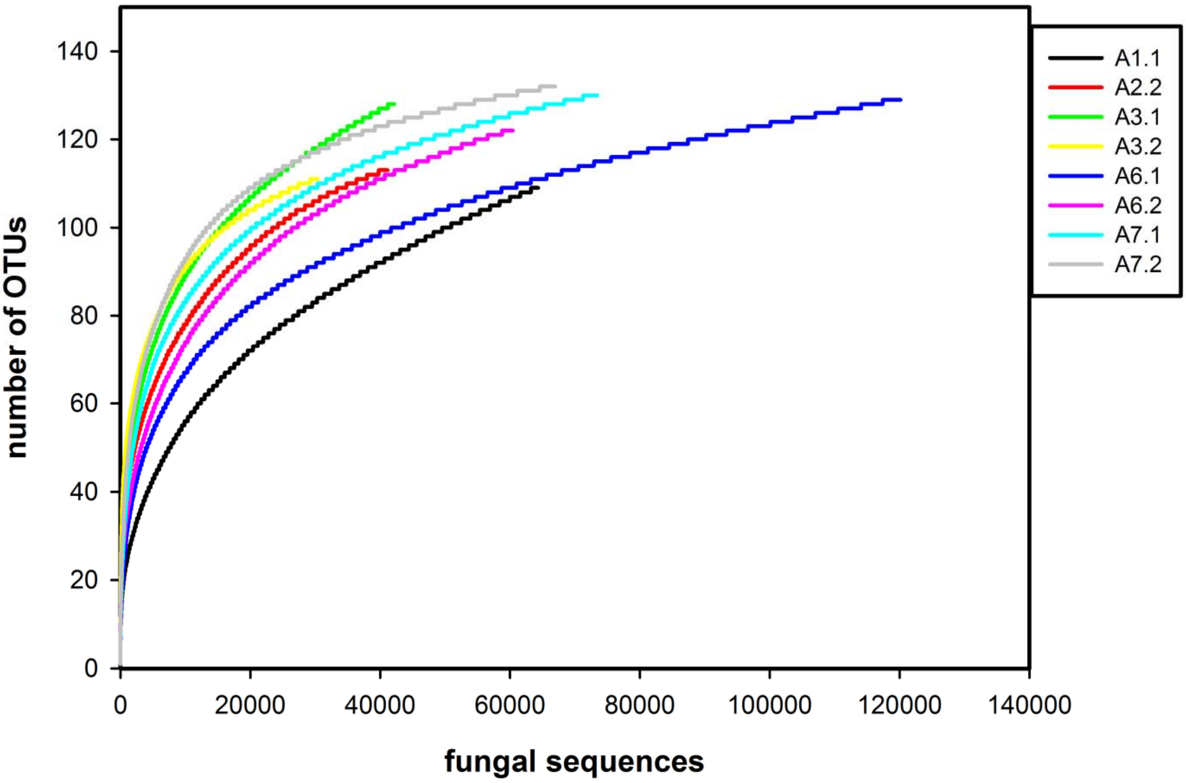

Fig. S5 Cluster analysis based on the distribution of culturable bacterial OTUs at the four sampling locations (A2, A3, A6, A7), generated by UPGMA. Values at branches are AU (approximately unbiased) p-values (left), BP (bootstrap probability) values (right), and cluster labels (bottom).

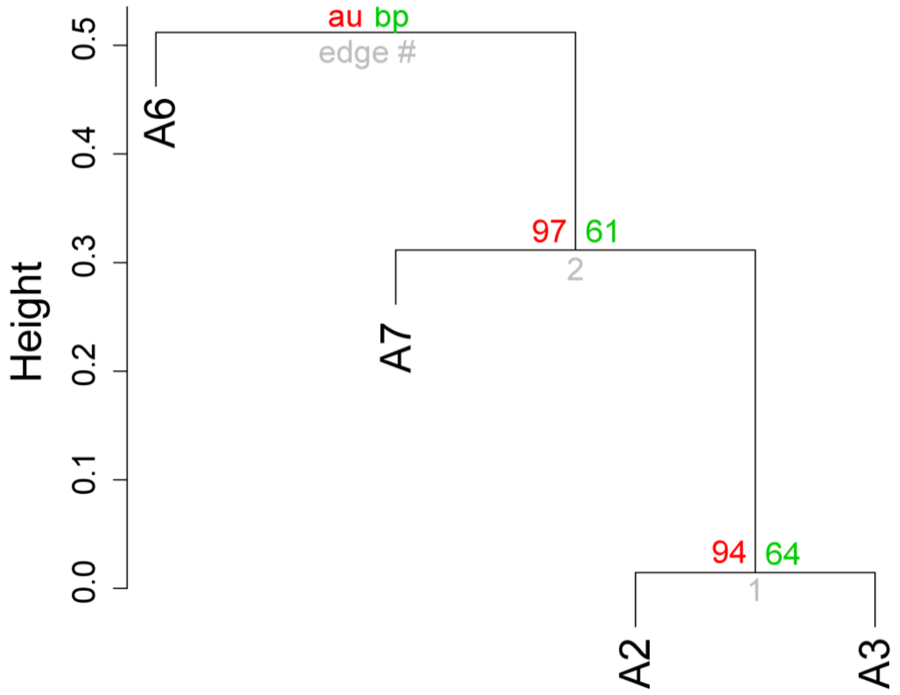

Supplement: Supplementary file 1 — (PDF 467 kb) [file 248_2016_904_MOESM1_ESM.pdf]
